# Clinical impact and predictive value of patient specific pre-bent rods in spinal deformity surgery: A comparative analysis of preoperative planned and postoperative outcomes

**DOI:** 10.1016/j.bas.2026.106068

**Published:** 2026-04-22

**Authors:** Zimo Lu, Jun Ao, Alexander Hammer, Denis Rappert, Olga Cheremina, Thomas Tischer, Christoph Lutter, Klaus John Schnake

**Affiliations:** a Department of Orthopedic Surgery, Zunyi Medical University, ZunYi, China; b Center for Spinal and Scoliosis Surgery, Malteser Waldkrankenhaus St. Marien, Erlangen, Germany; c Department of Orthopedic Surgery, University Medicine Rostock, Rostock, Germany; d Department of Neurosurgery, Paracelsus Medical University, Breslauer Straße 201, 90471, Nuremberg, Germany; e Department for Orthopaedic and Trauma, SurgeryMalteser Waldkrankenhaus St. Marien, Erlangen, Germany; f Department of Orthopedics and Traumatology, Paracelsus Private Medical University, Nuremberg, Germany

**Keywords:** Patient-specific rods, Adult spinal deformity, Sagittal alignment, Correction achievement ratio, Correction maintenance index, Proximal junctional kyphosis, Learning curve

## Abstract

**Introduction:**

Patient-specific rods (PSRs) represent an emerging technology for spinal deformity correction, offering personalized surgical planning based on individual anatomy.

**Research question:**

How accurately do PSRs achieve planned sagittal alignment corrections, and what factors influence surgical outcomes and complications?

**Material and methods:**

Twenty-two consecutive patients underwent spinal deformity correction using UNiD™ PSRs. Two novel metrics were developed: Correction Achievement Ratio (CAR) and Correction Maintenance Index (CMI). Radiographic parameters were measured preoperatively, postoperatively, and at one-year follow-up.

**Results:**

Twenty patients (91%) completed follow-up. PI-LL mismatch (CAR = 102.8%) and TPA (CAR = 91.9%) achieved near-perfect correction. PT (CAR = 149.2%) and SS (CAR = 123.5%) showed overcorrection. Learning curve analysis revealed improved accuracy after case 8. Overcorrection significantly increased complication risk (PT>120%: OR = 3.41, p = 0.025; PI-LL>120%: OR = 4.25, p = 0.007).

**Discussion and conclusion:**

PSRs effectively achieve planned sagittal corrections with high initial accuracy. However, maintaining correction remains challenging, and overcorrection increases complication risk. Surgical precision improves with experience. While promising for reducing mechanical complications, the technology requires further refinement for optimal outcomes.

## Highlights

• **Novel** **Metrics:** Correction Achievement Ratio (CAR) and Correction Maintenance Index (CMI) to evaluate accuracy and long-term stability.
• **Learning Curve:** Surgical accuracy with patient-specific rods significantly improves after approximately 8 cases.
• **Parameter-Specific Outcomes:** PI-LL mismatch and TPA achieved closest match to planned targets.
• **Risk Factors:** Overcorrection of PT and PI-LL mismatch significantly increases complication risk, particularly proximal junctional kyphosis.
• **Clinical** **Implications:** Patient-specific rods improve sagittal alignment, but maintaining long-term correction remains challenging.

## 1 Introduction

Spinal deformities greatly affect patients' quality of life by causing chronic pain, mobility limitation and even psychosocial burden (Kim et al., 2022). Spinal surgical correction aims to correct spinal alignment, relieve symptoms and improve functional outcomes and patient satisfaction. However, it is challenging to achieve consistent surgical correction because of the individual variability of patient's anatomy and the variations of surgical technique. In addition, outcome prediction remains difficult (Wang et al., 2015).

Recently, patient-specific rod (PSR) systems such as UNiD™ technology have been introduced in spinal deformity correction (Picton et al., 2024). PSR systems can be used for preoperative planning and manufacturing of rods tailored to each patient's anatomy. It is hoped that the use of PSR systems will improve surgical accuracy and decrease intraoperative surgical corrections. Research has shown that PSR systems can improve sagittal alignment accuracy and closely match preoperative planning targets (Prost et al., 2020a), reduce proximal junctional failures (Faulks et al., 2023), and obtain better alignment effects compared with rods (Nasto et al., 2025).

Although promising results have been reported, the detailed comparative data between the preoperative planning and the actual postoperative outcome are not available in the literature**.** Very few studies investigate the learning curve of adopting the PSR system and the correlations between alignment correction and postoperative complications such as rod fractures and junctional failures are explored. Surgical experience and accurate rod contouring may affect the outcome and complication rates (Dehghani et al., 2022). Accurate corrections and suboptimal ones may result from improper rod contouring without adequate information and guidance (Sardi et al., 2020).

Therefore, the primary objective of this study was to test these hypotheses by evaluating the accuracy of sagittal alignment correction achieved with PSR technology. Secondary objectives included characterizing the learning curve associated with PSR adoption, quantifying the relationship between correction magnitude and complication rates, and identifying preoperative parameters that predict surgical outcomes. To achieve these objectives, we developed two novel metrics: the Correction Achievement Ratio (CAR) to quantify surgical accuracy, and the Correction Maintenance Index (CMI) to assess the stability of correction over time.

## 2 Methods

This retrospective cohort study analyzed twenty-two consecutive patients who underwent spinal deformity correction using the UNiD™ rod system (Medtronic, Ireland) at our institution between October 2022 and March 2024. Although patient data were collected retrospectively from medical records and imaging databases, all radiographic measurements and clinical assessments had been performed prospectively as part of our standard clinical protocol for spinal deformity patients. This standardized protocol ensured consistent data collection across all patients, including preoperative planning parameters, immediate postoperative measurements, and follow-up assessments at predetermined intervals. Inclusion criteria were thoracolumbar adult deformity and an instrumented fusion of a minimum of 4 segments.

The study protocol was approved by the local Ethics Committee (23030). All patients provided an informed consent form in accordance with the Declaration of Helsinki.

All surgical procedures were performed by the same surgical team and UNiD™ patient specific rod system was used. Preoperative planning was done with the proprietary software of the manufacturer, and the target alignment parameters were calculated based on the patient's anatomy and published age-adjusted alignment goals.

All procedures were performed via a posterior approach with posterior pedicle screw instrumentation using the UNiD™ patient-specific rod system (Medtronic, Ireland). Moreover, the significant findings may suggest that S2-alar-iliac (S2AI) iliac screw fixation could indicate a critical technical consideration, as it was employed in 18 of 20 patients (90%), selected based on the extent of distal instrumentation, pelvic obliquity, and the surgical team's judgment regarding distal construct stability. Furthermore, the evidence may demonstrate that anterior column support appears to have been provided in 17 of 20 patients (85%): anterior lumbar interbody fusion (ALIF) was performed in 12 patients (60%), lateral lumbar interbody fusion (XLIF/LLIF) in 2 patients (10%), and transforaminal lumbar interbody fusion (TLIF) in 3 patients (15%), while 3 patients (15%) underwent posterior instrumentation without interbody fusion. Given that the results could indicate that posterior release is a key surgical objective, the significant data may suggest that posterior column osteotomies (PCO/Ponte osteotomies) were performed in 19 of 20 patients (95%) to achieve adequate posterior release. Pedicle subtraction osteotomy (PSO) shows severe fixed sagittal deformity addressed in 2 patients (10%). However, the important findings may indicate that decompression at stenotic levels appears to have been performed as clinically indicated, suggesting that the results could demonstrate a patient-specific approach to managing relevant surgical complexity.

Standard full spine standing radiographs were taken at four different time points:

● Preoperative baseline (Pre-Op)
● Preoperative planned parameters (Plan) - generated by UNiD™ planning software.
● Immediate postoperative (Post-Op) ≤ 2 weeks after surgery.
● One-year postoperative follow-up (1-Year) = 12 ± 1 months after surgery.

Sagittal alignment parameters were measured by two independent spine surgeons blinded to the clinical outcome and the interobserver reliability was calculated using the intraclass correlation coefficients (ICC) for the following sagittal alignment parameters:

● Pelvic Tilt (PT)
● Pelvic Incidence (PI)
● Sacral Slope (SS)
● Lumbar Lordosis (LL)
● Pelvic Incidence-Lumbar Lordosis mismatch (PI-LL)
● T1 Pelvic Angle (TPA)
● Sagittal Vertical Axis (SVA)

We documented postoperative complications, especially proximal junctional kyphosis (PJK) and need for revision surgery. Revision surgeries during the one-year follow-up were recorded and their indication was documented.

### 2.1 Statistical analysis

Statistical analyses were designed to test our primary hypotheses. For the first hypothesis regarding correction accuracy, one-sample t-tests were used to compare mean CAR values against the hypothesized 90% threshold for PI-LL mismatch and TPA. For the second hypothesis concerning the learning curve, we employed change-point analysis using CUSUM methodology to identify the case number at which surgical performance significantly improved. For the third hypothesis linking overcorrection to complications, logistic regression models were constructed with CAR categories (≤100%, 100-120%, >120%) as the independent variable and complication occurrence as the dependent variable.

To assess the precision and sustainability of the surgical correction we developed two metrics:

Correction Achievement Ratio (CAR) - assessing how closely the postoperative result matches the preoperative plan: CAR = (Post-Op value - Pre-Op value)/(Plan value - Pre-Op value) × 100%

Interpretation:

● CAR = 100%: Perfect achievement of planned correction
● CAR >100%: Overcorrection relative to plan
● CAR <100%: Undercorrection relative to plan

To avoid statistical artifacts when the denominator value approaches zero, we defined a minimum threshold of 3° for angular parameters and of 5 mm for SVA. When the preoperative and planned value differed less than this threshold, the parameter was considered clinically unchanged and thus excluded from the CAR calculation if it did not achieve the planned value.

Correction Maintenance Index (CMI) - quantifying the stability of correction over time: CMI = (1-Year value - Post-Op value)/(Post-Op value - Pre-Op value) × 100% Interpretation:

● CMI = 0%: Perfect maintenance of immediate postoperative correction
● CMI >0%: Loss of correction over time
● CMI <0%: Additional correction gained over time

CMI values were classified as:

● High stability: CMI <10%
● Moderate stability: CMI between 10 and 20%
● Low stability: CMI >20%

Learning Curve Analysis.

To assess the learning curve of the surgeons with the UNiD™ system we have performed complementary analyses on the t learning series.

Chronological case analysis: The cases were analyzed as a time series, with CAR values for PI-LL and SVA plotted against case number.

Cumulative summation (CUSUM) analysis: We calculated CUSUM for deviations from ideal correction (CAR = 100%) to identify turning points in the learning curve.

Case complexity stratification**:** Cases were categorized by complexity using a composite score based on:

● Preoperative SVA magnitude (<50 mm, 50-100 mm, >100 mm)
● Magnitude of planned correction (Δ PI-LL <10°, 10-20°, >20°)
● Patient factors (age >70, BMI >30)

Logistic regression analysis was applied to investigate the relationships between postoperative complications (PJK and revision surgery) and accuracy of correction. ORs were calculated with 95% confidence interval for each parameter to detect the effects of each parameter on postoperative complication risks.

Continuous variables were shown as mean ± SD. Statistical significance was accepted for p < 0.05. Paired t-tests were used to compare parameters between two time points. When the data did not follow normal distribution (Shapiro-Wilk test of normality), non-parametric tests (Wilcoxon signed-rank test) were used. All statistical analyses were performed using SPSS Statistics version 27.0 (IBM Corp., Armonk, NY, USA).

## 3 Results

**Fig. 1:**
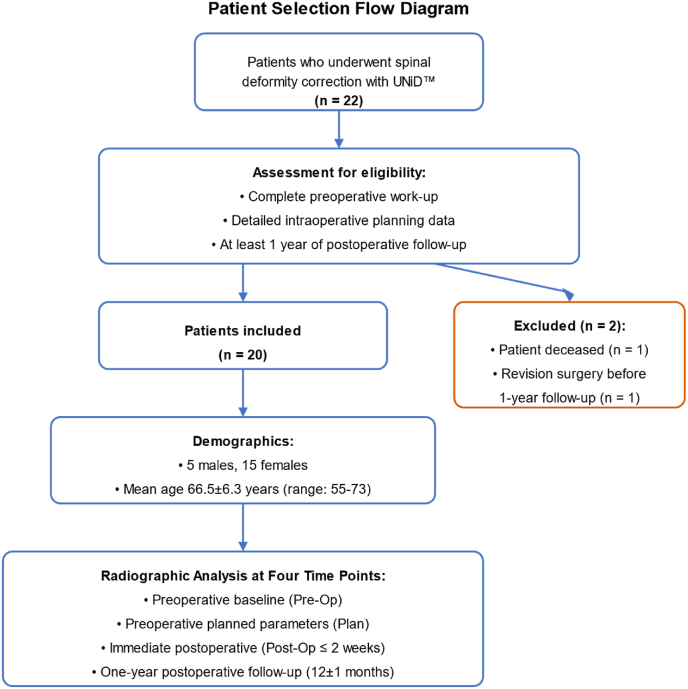
Flowchart patient selection.

Of the 22 patients initially enrolled, 20 were included in the study (6 males, 14 females; mean age, 66.5 ± 6.3 [range, 55–73] years; mean height, 166.3 ± 7.3 cm; and mean weight, 78.8 ± 17.2 kg) (Fig. 1).

Six patients (35%) developed complications within the one-year follow-up period: PJK was developed by 6 patients (30%), revision surgery was required in 6 patients (30%), surgical site infections developed in 2 patients (10%), and a dural tear developed in 1 patient (5%).

**Fig. 2:**
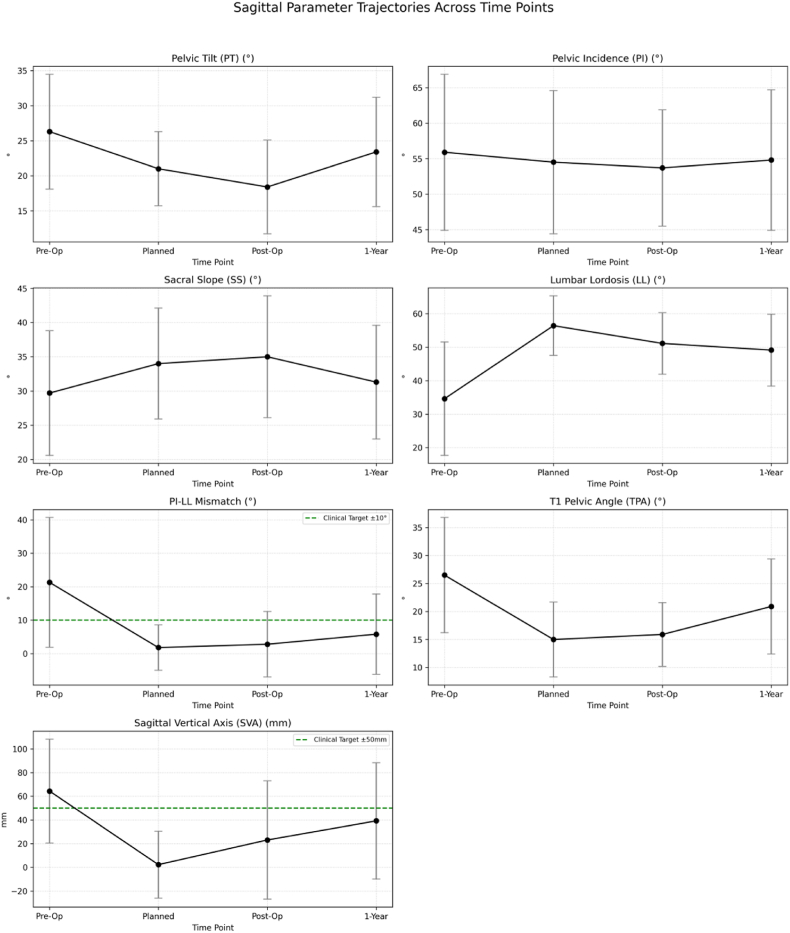
This figure shows the changes in key sagittal alignment parameters across four time points: preoperative baseline (Pre-Op), preoperative planned parameters (Plan), immediate postoperative (Post-Op), and one-year follow-up (1-Year). Parameters displayed include PT (Pelvic Tilt), SS (Sacral Slope), LL (Lumbar Lordosis), PI-LL mismatch (Pelvic Incidence minus Lumbar Lordosis), TPA (T1 Pelvic Angle), and SVA (Sagittal Vertical Axis).

Table 1 shows the sagittal alignment parameters at four different time points: preoperative, planned, immediate postoperative and one year follow up. There were significant improvements in several parameters between preoperative and immediate postoperative parameters. Correction in the preoperative to immediate postoperative period was very good with all parameters improving. There was regression of most parameters between the immediate postoperative period and one-year follow-up (some loss of correction) (PT increased from 18.4° to 23.4° and SVA increased from from 30.4 mm to 39.2 mm). The trend of each parameter over the four time points shows an initial improvement followed by some regression over time (Fig. 2).

**Table 1:** Sagittal alignment parameters at different time points (mean ± SD).^a^

Parameter	Preoperative	Planned	Immediate Postoperative	1-Year Follow-up	p-value^a^
PT (°)	26.3 ± 8.2	21.0 ± 5.3	18.4 ± 6.7	23.4 ± 7.8	<0.001
PI (°)	55.9 ± 11.0	54.5 ± 10.1	53.7 ± 8.2	54.8 ± 9.9	0.087
SS (°)	29.7 ± 9.1	34.0 ± 8.1	35.0 ± 8.9	31.3 ± 8.3	0.003
LL (°)	−34.6 ± 16.9	−56.4 ± 8.9	−51.1 ± 9.2	−49.1 ± 10.7	<0.001
PI-LL mismatch (°)	21.3 ± 19.4	−1.8 ± 6.8	2.8 ± 9.8	5.8 ± 12.0	<0.001
TPA (°)	26.5 ± 10.3	15.0 ± 6.7	15.9 ± 5.7	20.9 ± 8.5	<0.001
SVA (mm)	64.3 ± 43.9	2.3 ± 28.3	30.4 ± 45.5	39.2 ± 49.1	<0.001

Legend.

PT = pelvic tilt, PI = pelvic incidence, SS = sacral slope, LL = lumbar lordosis, TPA = T1 pelvic angle, SVA = sagittal vertical axis

a p < 0.05 for repeated measures ANOVA between preoperative, immediate postoperative and 1-year follow up values.

Table 2 displays that PT and SS tended to overcorrect, whereas LL and SVA tended to be undercorrected, relative to their planned values. The PI-LL mismatch and TPA achieved nearer to their planned targets, with CAR = 102.8% and 91.9% respectively.

**Table 2:** Correction achievement ratio (CAR) for sagittal alignment parameters.

Parameter	CAR (%) Mean ± SD	Interpretation
PT (°)	149.2 ± 114.5	Overcorrection
PI (°)	N/A^a^	Not applicable^a^
SS (°)	123.5 ± 89.3	Overcorrection
LL (°)	75.8 ± 38.6	Undercorrection
PI-LL mismatch (°)	102.8 ± 67.4	Slight overcorrection
TPA (°)	91.9 ± 39.3	Near target
SVA (mm)	81.5 ± 48.9	Undercorrection

a PI is an anatomical parameter that is not expected to change with surgery; hence, CAR could not be calculated.

The CMI analysis examined the stability of the surgical correction over the one-year follow-up period. Table 3 shows the CMI values, and stability classification, for each parameter. All parameters displayed low stability over time (CMI >20%). LL presented moderate stability (CMI = 12.5 ± 25.7%). High CMI for PT (63.2%) and SS (69.8%) suggested a high loss of correction during follow up.

**Table 3:** Correction maintenance Index (CMI) for sagittal alignment parameters.

Parameter	CMI (%) Mean ± SD	Stability Level
PT (°)	63.2 ± 58.9	Low
PI (°)	N/A^a^	Not applicable^a^
SS (°)	69.8 ± 85.4	Low
LL (°)	12.5 ± 25.7	Moderate
PI-LL mismatch (°)	25.4 ± 48.9	Low
TPA (°)	47.1 ± 53.5	Low
SVA (mm)	39.2 ± 65.8	Low

a PI is an anatomical parameter not expected to change significantly with surgery; therefore, CMI was not calculated.

The study may suggest that the mechanisms underlying low CMI stability could indicate a characteristic pattern of progressive proximal lumbar lordosis loss. However, the significant findings demonstrate that all 6 patients (30%) who developed PJK showed this pattern at the uppermost instrumented level, identified on serial radiographs as the dominant failure mechanism. Moreover, the evidence may indicate that no cases of pedicle screw loosening were identified at the pelvic fixation level. In light of these results, it could appear that both patients without S2AI fixation (10%) did not develop PJK during the follow-up period. Findings show no rod fractures observed throughout one-year follow-up.

**Fig. 3:**
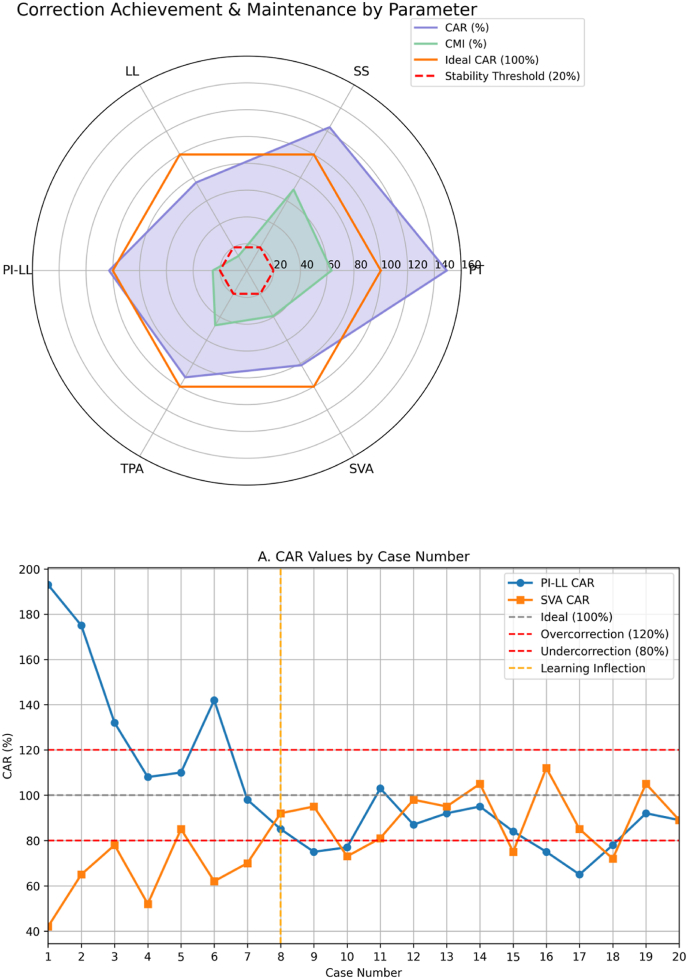
Radar chart showing the relationship between Correction Achievement Ratio (CAR) and Correction Maintenance Index (CMI) for sagittal alignment parameters in spinal deformity correction. Parameters included: Pelvic Tilt (PT), Sacral Slope (SS), Lumbar Lordosis (LL), Pelvic Incidence-Lumbar Lordosis mismatch (PI-LL), T1 Pelvic Angle (TPA), and Sagittal Vertical Axis (SVA). Values closer to 100% for CAR indicate optimal correction, while lower CMI values represent better maintenance of correction at one-year follow-up.

Fig. 3 radar chart illustrates the relationship between CAR and CMI among parameters, showing the pattern between degree of correction achievement and ability to maintain the correction.

**Fig. 4:**
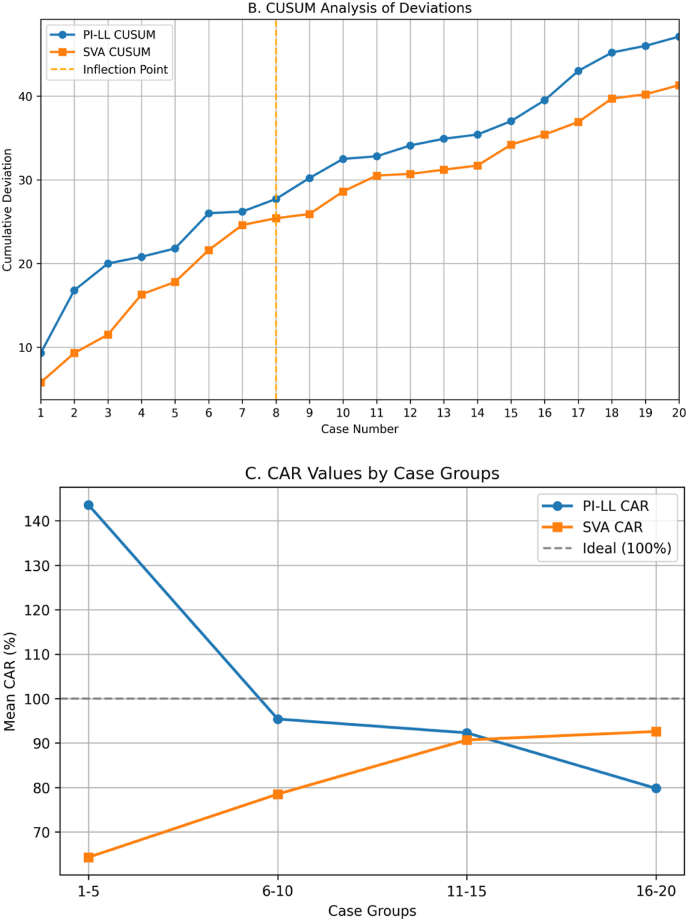
A (CAR by Case): Correction Achievement Ratio (CAR) for PI-LL mismatch and SVA plotted by consecutive case number. Fig. 4B (CUSUM Analysis): Cumulative Summation analysis showing deviations from ideal correction (CAR = 100%). Fig. 4C (CAR by Groups): Progression of correction accuracy by groups of cases (cases 1-5, 6-10, 11-15, and 16-20).

Chronological analysis of consecutive cases showed an evolving pattern of surgical skill with the UNiD™ system. Fig. 4 illustrates the CAR of PI-LL mismatch and SVA plotted as a function of case number. CUSUM analysis showed a significant turning point around case 8 and a drastic reduction in variability after that point, especially for PI-LL mismatch. Table 4 illustrates the CAR analysis by case groups.

**Table 4:** Correction achievement ratio (CAR) values for PI-LL and SVA by consecutive case groups.

Patient Group	Number of Cases	PI-LL CAR (%) Mean ± SD	SVA CAR (%) Mean ± SD
1-5	5	143.6 ± 98.7	64.3 ± 51.8
6-10	5	95.4 ± 62.9	78.5 ± 59.2
11-15	5	92.3 ± 38.1	90.7 ± 35.4
16-20	5	79.8 ± 45.3	92.6 ± 49.2

Fig. 4B CUSUM analysis illustrates the learning curve. The slope of the curve decreased noticeably after case 8. The analysis revealed an initial overcorrection period (cases 1-5), followed by successive correction towards the planned targets. Starting with cases 11-15, the CAR values of PI-LL and SVA approached the ideal target (100%). In the last group of cases (cases 16-20), there was a tendency towards undercorrection of PI-LL (79.8%) while optimal correction was achieved for SVA (92.6%).

Analysis of logistic regression was performed to explore associations between correction accuracy and postoperative complications [Table 5]. A visually shows the risk ratios and the statistical significance of these risk ratios of the respective parameters and indicates that PI-LL is the most significant parameter regarding the risk of complications. As shown in [Table 5], overcorrection of PT (CAR >120%) and PI-LL mismatch (CAR >120%) were significantly associated with an increased risk of complications. Complication risk was significantly higher in the PJK group. When stratified according to case complexity, high complexity ASD cases (defined as preoperative SVA >100 mm or planned ΔPI-LL >20°) showed a significantly higher complication rate (58.3%) as compared with medium complexity and low complexity cases (25.0% and 0%, respectively; p = 0.021) [Fig. 4B]. Table 5 illustrates the PI-LL CAR values and PJK occurrence. The results indicate a markedly high-risk ratio of PJK in patients with CAR >120%.

**Table 5:** Logistic regression analysis for complication risk factors.

Parameter	Odds Ratio (95% CI)	p-value
CAR PT (>120%)	3.41 (1.17-9.95)	0.025
CAR LL (<80%)	1.83 (0.64-5.27)	0.261
CAR PI-LL (>120%)	4.25 (1.48-12.21)	0.007
CAR TPA (<80%)	2.02 (0.73-5.61)	0.178
CAR SVA (<80%)	1.55 (0.52-4.65)	0.433

## 4 Discussion

This study evaluated the accuracy of PSR alignment parameters achieved at surgery, analyzed the learning curve, and investigated the correlation between the degree of alignment correction and complication rates.

We developed specific metrics—Correction Achievement Ratio (CAR) and Correction Maintenance Index (CMI)—to evaluate the clinical performance of PSR in ASD surgery.

Our findings provide evidence to support our hypotheses regarding the performance of patient-specific rod systems in adult spinal deformity surgery. Our first hypothesis was partially supported: while TPA achieved a CAR of 91.9%, closely meeting our hypothesized 90% threshold, PI-LL mismatch slightly exceeded expectations at 102.8%. These findings suggest that PSR technology can achieve high accuracy for certain parameters, though the degree of accuracy varies by specific measurement.

Our second hypothesis regarding the learning curve was supported but with an important modification. The significant improvement in surgical accuracy occurred at case 8 rather than case 10, indicating a steeper learning curve than anticipated. This earlier proficiency point has important implications for surgical training and implementation protocols.

Most notably, our third hypothesis was strongly supported by the data. Overcorrection beyond 120% of planned targets was significantly associated with increased complication risk, with odds ratios of 3.41 for PT overcorrection and 4.25 for PI-LL overcorrection. This finding underscores the importance of precise preoperative planning and avoiding the temptation to overcorrect deformities.

Our results show that sagittal parameters improved after application of UNiD™ patient-specific pre-bent rods. Especially the PT improved significantly from a preoperative 26° to 18° at the postoperative follow-up which agrees with the reported improvement in PT from 24° to 18° with PSR(Sadrameli et al., 2020). Similarly, SVA improved from a preoperative 67 mm to 29 mm at the postoperative follow-up, which was consistent with previously reported results (preoperative 65 mm versus postoperative 38 mm) (Prost et al., 2020a).

However, we also found a loss of correction. At one-year follow-up, SVA increased from postoperative 29 mm to 40 mm (CMI = 39.2%) and PT rebounded from 18° to 23° (CMI = 63.2%). Prost et al. also showed a time-dependent loss of correction, with 11 cases (12.8%) presenting with an increase in SVA at one-year follow-up out of 86 patients with one-year follow-up (Prost et al., 2020b). Therefore, although patient-specific rods can obtain good correction, they seem to be difficult to maintain in the long term.

Notably, our results are different from Faulks et al. They reported lower proximal junctional failure rate (5% versus 35% reported in the literature) in 20 patients with patient specific rods (Faulks et al., 2023). Our PJK incidence was 30% as compared to 50%+ in traditional rod curves reports (McDonnell et al., 2021). These differences may be related to the overall patient age, degree of preoperative deformity, and surgical technique among other factors between cohorts.

The results of our study suggests that while patient-specific rods can achieve the planned correction target, especially for PI-LL (CAR = 102.8%), challenges remain in correcting PT and SVA, with CAR value of 149.2% (over correction) and 81.5% (under correction), respectively. Similar results were reported by Ou-Yang et al. who indicated the existence of significant gaps between preoperative plans and postoperative results even when using patient specific rods (Ou et al., 2022).

In contrast, Sadrameli et al. reported no statistically significant differences between planned and postoperative results (Sadrameli et al., 2020), while Kleck et al. indicated that, although SVA planning and postoperative results were very close, the predictive value was limited as sagittal balance correction occurred more frequently than expected (R^2^ = 0.05-0.36) (Kleck et al., 2020). These differences may represent the differences in planning software and prediction among the studies. These results also indicate the limited understanding of the interactions among thoracic and pelvic parameters.

As mentioned by Barton et al., the possible reasons for such differences may be existence of 4 major factors deviating the corrections from the surgical plan: (1) deviation from the surgical plan, (2) planned osteotomy angles are not achieved, (3) inability to correlate proximal junctional kyphosis and reciprocal kyphosis to postoperative kyphosis and (4) difficulty in predicting postoperative pelvic relaxation (Barton et al., 2016). Therefore, it is important to keep improving the prediction model with machine learning approach as one tried in newest versions of UNiD™ Adaptive Spine Intelligence system.

In this study, we have developed CAR and CMI as new approaches to assess spinal deformity correction. While simple parameters comparison has been used conventionally, CAR provides the amount of planned target achievement in a numerical value. CMI, on the other hand, represents the degree of correction maintenance through the follow-up. While planned targets were initially overcorrected (CAR>120%), PT and SS demonstrated poor long-term stability (CMI>60%). In contrast, LL, which was undercorrected (CAR = 75.8%), demonstrated better maintenance (CMI = 12.5).

The assessment approach in this study goes beyond the simple pre-post comparisons used by other authors (Sadrameli et al., 2020)^,^ (Solla et al., 2019). It includes not only the amount of postoperative improvement, but also the degree of matching with planned targets and long-term stability. This comprehensive assessment approach will be important to gain better understanding of advantages and limitations of patient-specific rod technology. In this regard, in Fig. 2, radar chart displays the CAR and CMI variations through parameters, we think the readers can easily understand the trends of overcorrection versus undercorrection, and the stability levels.

Our logistic regression analysis demonstrated that PT overcorrection (CAR>120%) and PI-LL overcorrection (CAR>120%) were significantly associated with complication risk, especially PJK (OR = 3.41, p = 0.025 and OR = 4.25, p = 0.007, respectively). Faulks et al. also reported that overcorrection may increase the stress and failure at adjacent segments (Faulks et al., 2023).

Encouragingly, we did not observe any rod breakages during follow-up time within our study similar to reported rod fracture rates with patient-specific rods (2.2% versus 15% with conventional rods)(Fiere et al., 2016). This might be due to a reduction of intraoperative bending microfractures and therefore an increase of the rod fatigue life.

In agreement with Thomas et al., we could show that patient-specific rods improved radiological parameters but did not prevent PJK (Thomas et al., 2023). Therefore, besides an optimal rod geometry, other parameters such as bone quality or the selection of fusion level might be of major importance for complication prevention.

Our stratified analysis may suggest that progressive proximal lumbar lordosis loss leading to PJK could indicate the dominant mechanism of correction loss in this cohort, consistent with the high CMI values observed for PT (63.2%) and SS (69.8%). Moreover, the mechanical transition from a rigid instrumented construct to the mobile native suprajacent segments might indicate that stress concentration at the proximal junction, in the absence of sufficient anterior column support or optimal rod geometry, could demonstrate a predisposition to progressive kyphotic failure. Furthermore, the significant findings may suggest that all 6 PJK cases occurred in patients who had received anterior column support (ALIF or TLIF), indicating that interbody fusion alone appears insufficient to prevent PJK when proximal junctional biomechanics are unfavourable. In light of the evidence, the results could indicate that PSR technology optimises rod geometry and reduces intraoperative bending microfractures, but does not eliminate the fundamental biomechanical vulnerabilities at the proximal junction. Concurrent optimisation of fusion level selection, proximal rod contouring strategy, and pedicle screw density at the uppermost instrumented vertebra may reduce PJK risk.

Our study shows that with increasing surgical experience, PI-LL CAR improved from 143% (overcorrection) over correction in the first 5 cases to 92% (close to 100%) in cases 11-15. This indicates that there is a learning curve effect, but it is different to the effect shown with the system familiarization as emphasized by Ou-Yang et al. (Ou et al., 2022). This learning process as illustrated in Fig. 4 shows a substantial increase in correction accuracy with increasing surgical experience. Using the CUSUM analysis, a significant inflection at case 8 is observed beyond which the surgeons were significantly more consistent. The final group of cases (cases 16-20) demonstrated a reduction in PI-LL CAR to 79% (undercorrection), which may indicate a trend toward handling more difficult cases as Kleck et al. (2020) observed handling more complex cases as self-confidence increased. Therefore, emphasis on surgical planning accuracy should not be relaxed even after mastery is obtained.

Several limitations should be acknowledged. First, the retrospective nature of this study introduces potential selection bias and limits our ability to control for all confounding variables. Although clinical and radiographic data were collected using standardized protocols, the lack of prospective randomization prevents definitive causal conclusions. Second, while our study was hypothesis-driven, the relatively small sample size of 20 patients limits the statistical power for some analyses, particularly subgroup comparisons. Future prospective studies with larger cohorts are needed to validate these findings and refine our understanding of optimal correction targets.

## 5 Conclusion

Patient-specific rods effectively achieve planned sagittal alignment corrections with high initial accuracy. The technology shows promise in reducing mechanical complications, particularly rod fractures. However, maintaining long-term correction remains challenging. Importantly, overcorrection significantly increases complication risks, emphasizing the need for precise preoperative planning. While PSR technology represents an advancement in spinal deformity surgery, further refinement is needed to optimize outcomes.

## Funding

Financial support from the program of China Scholarships Council (No.202308520054).

## Declaration of competing interest

The authors declare the following financial interests/personal relationships which may be considered as potential competing interests:

Alexander Hammer reports financial support was provided by Medtronic. Denis Rappert reports financial support was provided by Medtronic. Klaus John Schnake reports financial support was provided by Medtronic. Zimo Lu reports statistical analysis and writing assistance were provided by Medtronic. Olga Cheremina reports statistical analysis was provided by Medtronic. If there are other authors, they declare that they have no known competing financial interests or personal relationships that could have appeared to influence the work reported in this paper.
